# Formative key feature examinations as innovative teaching approach in dental education: A project report

**DOI:** 10.3205/zma001694

**Published:** 2024-09-16

**Authors:** Tim Becker, Marc André Ackermann, Sabine Sennhenn-Kirchner

**Affiliations:** 1 University Medical Center Göttingen, Study Deanery of the Medical Faculty, Division of Medical Education, Göttingen, Germany; 2 University Medical Center Göttingen, Department of Oral and Maxillofacial Surgery, Göttingen, Germany

**Keywords:** dental education, dental licensing regulations, key feature cases, formative examination, test-enhanced learning, clinical reasoning, clinical decision-making, virtual patients, digital teaching, e-learning

## Abstract

**Introduction:**

Clinical reasoning ability is one of the core competencies of physicians. It should already be trained during undergraduate medical education. At University Medical Center Göttingen (UMG), medical students can participate in formative key feature examinations in which they work on virtual patient cases in order to apply and deepen the procedural knowledge acquired in lectures and seminars.

**Problem and objective:**

While this teaching format is already established in the *medica*l curriculum at the UMG, it has not yet been implemented in the *dental *curriculum at the same institution. Therefore, the current project aimed to evaluate the feasibility of formative key feature examinations in dental education.

**Methods:**

In 2022, new key feature cases focusing on dental-surgical teaching content were created. For pilot testing, the new cases were worked on by two cohorts of dental students via an online learning platform in February 2023. The students were also asked to complete an anonymous online questionnaire in order to evaluate the new teaching format.

**Results:**

Overall, the formative key feature examinations were evaluated positively by the dental students, and they demanded for further dental key feature cases. However, descriptive analyses of item characteristics as well as students’ comments in the questionnaire revealed some potential for improvements, so that a few cases were partly revised afterwards.

**Conclusion and outlook:**

This project shows that formative key feature examinations are feasible in dental education and that dental students can benefit from working on virtual case scenarios. Whether dental students’ clinical reasoning competence can be improved by completing formative key feature examinations is being investigated in an ongoing study at the UMG.

## Zusammenfassung

**Einleitung:**

Klinische Entscheidungskompetenz gehört zu den Kernkompetenzen von Mediziner*innen. Sie sollte bereits während des Studiums trainiert werden. An der Universitätsmedizin Göttingen (UMG) können Studierende der Humanmedizin bereits seit 2013 an formativen Key-Feature-Prüfungen teilnehmen, um das in Vorlesungen und Seminaren erworbene prozedurale Wissen in virtuellen klinischen Fällen anzuwenden und zu vertiefen.

**Problem- und Zielstellung:**

Während das Lehrformat im Humanmedizinstudium an der UMG bereits etabliert ist, wurde es im Studium der Zahnmedizin am gleichen Standort bislang nicht implementiert. Das hier vorgestellte Projekt zielte deshalb darauf ab, die Realisierbarkeit formativer Key-Feature-Prüfungen in der Zahnmedizin zu evaluieren.

**Methoden:**

Im Sommer und Herbst 2022 wurden zahnmedizinisch-chirurgische Key-Feature-Fälle erstellt. Zur Pilotierung wurden die virtuellen Fälle zwei Kohorten von Zahnmedizinstudierenden über eine Online-Lernplattform zur Verfügung gestellt. Die Studierenden wurden außerdem gebeten, einen anonymen Online-Evaluationsfragebogen zu dem neuen Lehrformat auszufüllen.

**Ergebnisse:**

Die Evaluationsergebnisse zeigten, dass die formativen Key-Feature-Prüfungen von den Zahnmedizinstudierenden positiv bewertet wurden und dass die Studierenden sich weitere Fälle wünschten. Durch deskriptive Analysen der Fallbearbeitungen und die studentische Evaluation wurde jedoch auch Verbesserungspotential in den erstellten Key-Feature-Fällen sichtbar, so dass die Fälle im Nachgang teilweise überarbeitet wurden.

**Schlussfolgerung und Ausblick:**

Das vorliegende Projekt zeigt, dass formative Key-Feature-Prüfungen auch im Studium der Zahnmedizin umsetzbar sind und dass Studierende von der Bearbeitung der virtuellen Fälle profitieren können. Inwieweit sich die Bearbeitung von Key-Feature-Fällen objektiv messbar auf den Lernerfolg der Studierenden auswirkt, wird zurzeit in einer laufenden Studie untersucht.

## Introduction

### Theoretical background

One of the core competencies of physicians is the ability to arrive at correct diagnoses and treatment recommendations based on the results of appropriate history taking and diagnostic testing. Such complex cognitive processes are referred to as “clinical reasoning” and they should already be trained during undergraduate medical education [1], [2].

One effective teaching format to improve clinical reasoning competence is *case-based learning* in which medical students are faced with clinical problems using specific case examples [3]. However, case-based learning in small groups with *real* patients is resource-intensive and not easy to standardise [4]. Therefore, computer-assisted case-based learning using *virtual* patients is a suitable alternative since various digital case scenarios can be worked on by large groups of students in order to train clinical reasoning processes [5], [6].

Students’ performance regarding clinical reasoning can be assessed using so-called *key feature examinations*: within this test format, students are exposed to clinical case scenarios and they have to answer questions that focus on the critical steps (namely the *key features*) of these cases, such as diagnostic or therapeutic procedures [7], [8]. However, key feature examinations can be used not only for *assessing* but even for *improving *students’ clinical reasoning competence: this approach of *test-enhanced learning* is based on the so-called testing effect suggesting that repeated retrieval of memorised content (e.g. by taking formative tests) can stimulate cognitive processes and improve long-term retention of the retrieved content [9], [10].

### Formative key feature examinations at University Medical Center Göttingen

With this in mind, a specific teaching format was implemented at University Medical Center Göttingen (UMG) in the clinical phase of the medical curriculum in 2013: In weekly computer-assisted seminars, students can participate in formative key feature examinations in order to apply and deepen the procedural knowledge they gained in previous lectures and seminars. This specific teaching format has been accompanied by research, and several studies have shown that medical students’ clinical reasoning competence can be improved by completing formative key feature examinations [4], [11], [12], [13].

### Objectives of the present project

There is a need to train clinical reasoning processes not only in medical education but also in dental education [14]. However, while the teaching format of formative key feature examinations is already established in the *medical *curriculum at the UMG, it has not yet been implemented in the *dental* curriculum at the same institution. Therefore, the present project aimed to create new dental key feature cases and to evaluate their feasibility in dental education at the UMG. Another objective was to define a standardised procedure for creating further dental key feature cases. Overall, dental students should have the possibility to benefit from formative key feature examinations in the same way as medical students do at the UMG.

## Methods

### Creating dental key feature cases

**Figure 1:**
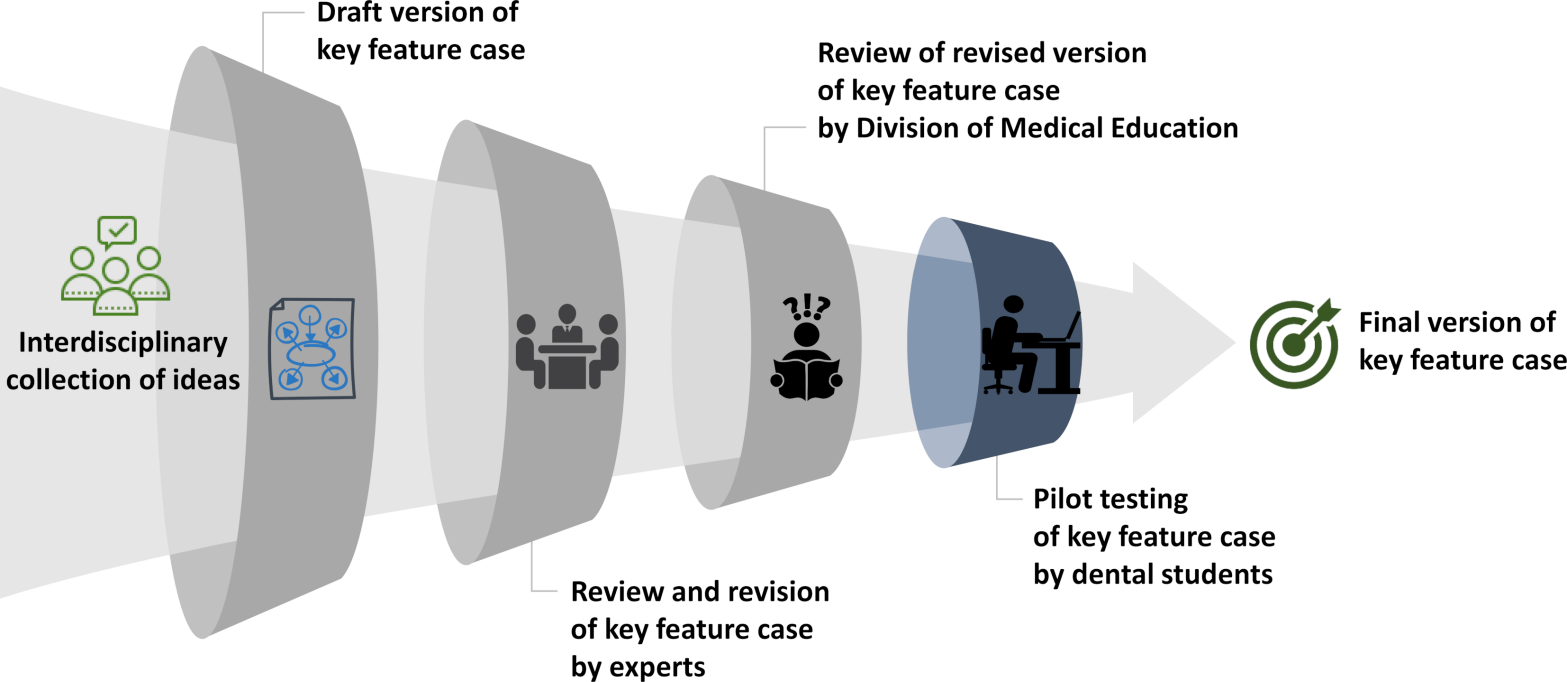
Standardised step-by-step procedure for creating dental key feature cases

In 2022, six initial key feature cases focusing on dental-surgical teaching content were created in interdisciplinary cooperation between the Division of Medical Education and the Department of Oral and Maxillofacial Surgery at the UMG. The step-by-step procedure for creating the new cases was based on the practical guide published by Kopp et al. [15]. However, it was complemented by repeated reviews and revisions by experts of the two institutions mentioned above (see figure 1 (Fig. 1)). This iterative process of creating cases was intended to ensure the highest possible quality of the dental key feature cases even before piloting. After finalisation, the new cases were transferred into the online learning platform ILIAS which is used for providing digital teaching resources at the UMG.

### Piloting and evaluating the new cases

**Figure 2:**
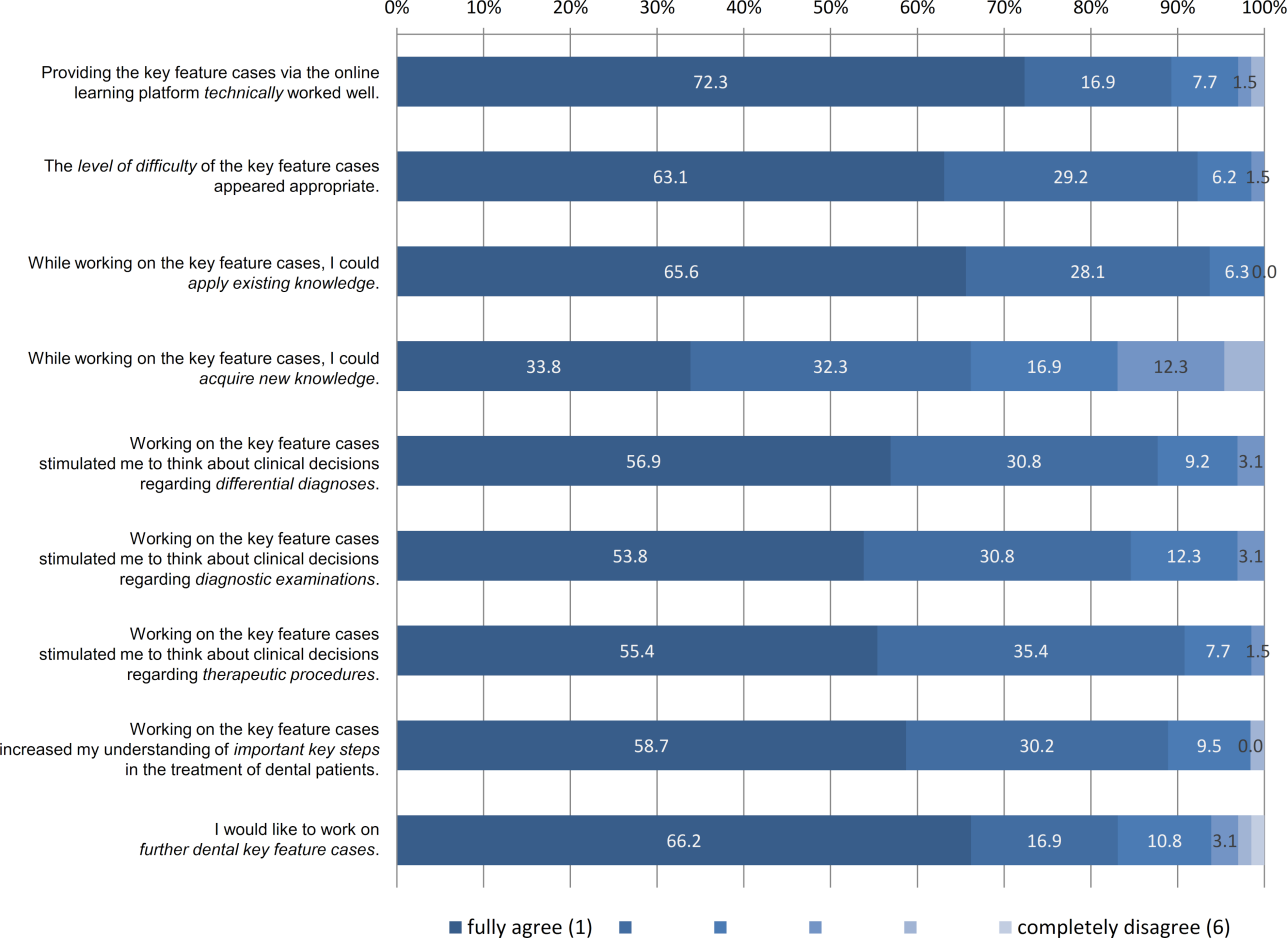
Proportions of students’ ratings on the closed items of the evaluation questionnaire

For pilot testing, three of the six new key feature cases were worked on by two cohorts of fourth year undergraduate dental students in computer-based seminars in February 2023. Additionally, the students were asked to evaluate the new teaching format by completing an online questionnaire using the evaluation software EvaSys. The anonymous questionnaire contained closed items (that could be rated on six-point scales, see figure 2 (Fig. 2)) as well as open-ended questions. Data collection and processing were approved by the ethics committee of the UMG (application no. 15/1/23). The students provided written consent to participate in the project.

After the students had completed working on the cases, data were exported from ILIAS and analysed anonymously. Descriptive analyses included students’ login times (i.e. the time required for completing the cases) as well as the percentages of correct and incorrect answers to the individual key feature questions. Additionally, analyses of item difficulty and item discrimination indices were performed [16] in order to assess the quality of the individual questions and the entire key feature cases and, if necessary, to make revisions. Data from the evaluation questionnaire were exported from EvaSys and analysed descriptively as well.

## Results

### Results of the key feature cases

The dental key feature cases were completed by 71 of the eligible 79 students (=89.9%). The average time required for completing the three cases was 15:56 (±5:21) minutes, and the students achieved a mean of 11.8 (±2.7) out of 16 points. Analyses of item characteristics showed that most of the key feature questions had adequate difficulty and discrimination indices. For two questions, however, the item characteristics as well as wide ranges of students’ answers indicated that these questions had not been formulated precisely enough and therefore needed to be revised. As an example, table 1 (Tab. 1) shows the item difficulty and discrimination indices for one of the three key feature cases (which is attached to this project report as attachment 1 ).

### Results of the evaluation questionnaire

The evaluation questionnaire was completed anonymously by 65 of the 71 students (=91.5%). Their ratings on the closed items showed, for example, that providing the key feature cases via the online platform worked well technically, and that working on the cases contributed to deal with dental teaching content and clinical decisions, and that students would appreciate the enhancement of the new teaching format (see figure 2 (Fig. 2)). Comments to the open-ended questions also showed students’ overall positive evaluation of the dental key feature cases as well as their demand for enhancing the teaching format. In addition to this general feedback, the comments also contained some specific suggestions for revising the key feature cases, such as adding more synonyms of correct answers (e.g. alternative spellings or common abbreviations) into the drop-down lists of the long menu format [15] which is used in the cases.

## Discussion and outlook

The present project aimed to evaluate the feasibility of formative key feature examinations in dental education, as this teaching format had not yet been implemented in the *dental *curriculum at the UMG (although being established for years in the *medical *curriculum at the same institution). Even internationally, it seems that only a few faculties use key feature cases in dental education: We are aware of only two studies in which key feature examinations were used to assess clinical reasoning competence of dental students [17], [18]. In one of these studies, Owlia et al. found that the clinical reasoning skills of 11^th^ semester students were at a low level and they concluded that there is a need to train clinical reasoning processes already during undergraduate dental education [18]. Thus, our concept of using key feature examinations not only for *assessing* but rather for *improving* students’ clinical reasoning competence (in the sense of test-enhanced learning) represents an innovative teaching approach in dental education. Moreover, the new teaching format complies with the new German dental licensing regulations which recommend to promote students’ problem-based learning using specific case scenarios.

Creating the new dental key feature cases was time-consuming. This was, on the one hand, due to the complex long menu format [15] and the detailed feedback texts (which were automatically displayed to the students after having answered a key feature question, see attachment 1 ). On the other hand, it was due to the step-by-step procedure of creating the cases using iterative reviews and revisions by various experts (see figure 1 (Fig. 1)). However, it can be assumed that a kind of learning curve occurs and results in a less time-consuming creation of further dental key feature cases. Besides that, the iterative process of creating cases ensures a high quality of new cases even before piloting (demonstrated by mostly adequate item difficulty and discrimination indices in our project) so that revisions of the new cases are less time-consuming as well.

During pilot testing, the new cases were positively evaluated by the students overall. Comments to the open-ended questions showed that students demanded for further dental key feature cases, as this teaching format provides the possibility to apply and deepen their procedural knowledge within specific clinical contexts. Only one closed item of the questionnaire, evaluating the acquisition of new knowledge by working on the key feature cases, was rated relatively lower than the rest of the items. However, this rating of the students fits well with the intention of the teaching format: since focusing on teaching content of previous lectures and seminars, the key feature cases aim at applying existing knowledge rather than acquiring new knowledge. Besides general feedback on satisfaction, the students also made some specific suggestions for revising the cases. Thus, the results of the evaluation questionnaire (as well as the descriptive analyses of item characteristics) played an important role in validating and optimising the new dental key feature cases [15], [19].

In conclusion, our project shows that formative key feature examinations are feasible in dental education and that dental students can benefit from working on virtual case scenarios. However, whether dental students’ clinical reasoning competence can be improved by completing formative key feature examinations is currently investigated in an ongoing study at the UMG.

## Notes

### Authorship

Tim Becker and Marc André Ackermann share the first authorship.

### Authors’ ORCIDs

- Tim Becker: [0009-0004-7139-7559]
- Marc André Ackermann: [0009-0005-8550-9352]

## Acknowledgements

The authors acknowledge support by the Open Access Publication Funds of the Göttingen University. And they would like to thank all dental students for participating in this project.

## Competing interests

The authors declare that they have no competing interests.

## Supplementary Material

Exemplary key feature case “management of oro-antral communication”

## Figures and Tables

**Table 1:**
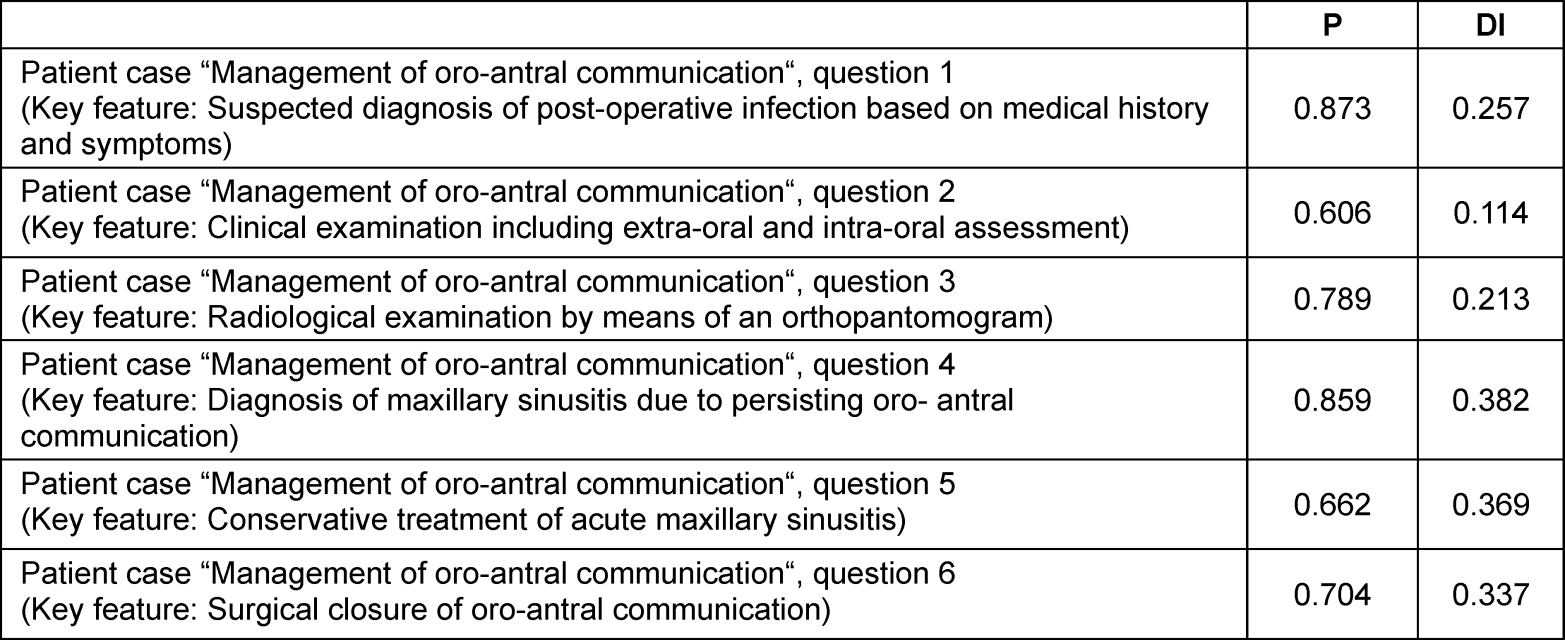
Item difficulty (P) and item discrimination (DI) indices of the key feature case “management of oro-antral communication” after being completed by 71 dental students during pilot testing in February 2023. For question 2, the low discrimination index (below 0.2) as well as a wide range of students’ answers led to a revision of this question.
