# Supplementary material for: Formative key feature examinations as innovative teaching approach in dental education: A project report
Source: GMS J Med Educ. 2024 Sep 16;41(4):Doc39. doi: 10.3205/zma001694 (PMC11474647; doi:10.3205/zma001694)
Supplement: Exemplary key feature case “management of oro-antral communication” [file JME-41-39-s-001.pdf]

Attachment 1: Exemplary key feature case "management of oro-antral communication"

**Authors:**	Dr. Marc André Ackermann, Tim Becker MME, PD Dr. Sabine Sennhenn-Kirchner MME
**Clinical problem:**	Management of oro-antral communication
**Key features of the clinical problem:**	1. Suspected diagnosis of post-operative infection based on medical history and symptoms<br>2. Clinical examination including extra-oral and intra-oral assessment<br>3. Radiological examination by means of an orthopantomogram<br>4. Diagnosis of maxillary sinusitis due to persisting oro-antral communication<br>5. Conservative treatment of acute maxillary sinusitis<br>6. Surgical closure of oro-antral communication

Case vignette:

A 41-year-old female patient comes to your dental emergency service complaining of discomfort in the right upper jaw and the right side of her face. The patient reports that a few days ago one of her molars was extracted by her family dentist. After the extraction, she initially only felt a tolerable soreness, but now the pain has increased significantly and can no longer be clearly localised to the wound of the extracted tooth. The patient feels a throbbing pain in the right side of her face, especially when bending over or changing the position of her head.
The patient's general medical history is unremarkable. She does not report any diseases or regular medication or relevant allergies.

Attachment 1 to Becker T, Ackermann MA, Sennhenn-Kirchner S. *Formative key feature examinations as innovative teaching approach in dental education: A project report.* GMS J Med Educ. 2024;41(4):Doc39.
DOI: 10.3205/zma001694

Question 1:

Based on the available information, which is the most likely working diagnosis?

Correct answers:	post-operative infection, wound infection, pain after extraction, dolor post extractionem, alveolar osteitis, dry socket, maxillary sinusitis, acute maxillary sinusitis, oro-antral communication, OAC, oro-maxillary sinus perforation
Distractors:	pulpitis, periodontitis in the first quadrant, midface fracture, zygomatic fracture, fractured alveolar process

Feedback:

This patient most likely suffers from a post-operative infection of the extraction wound, possibly in combination with an oro-antral communication.

Detailed feedback on question 1

In this case, the patient's extraction wound has possibly become infected due to inadequate primary wound healing. This would explain the increasing pain symptoms. Furthermore, a molar extraction in the maxilla carries the risk of developing an oro-maxillary sinus perforation (or oro-antral communication, OAC), so that an OAC with secondary infection of the ipsilateral maxillary sinus may be present here. The pain symptoms, which can no longer be clearly localized to the extraction wound, also indicate inflammatory involvement of surrounding structures.

Although pulpitis of the adjacent teeth and other inflammatory changes of the gingiva or periodontium are also possible, they are not the most likely cause of the symptoms described. Nevertheless, they should be excluded in the further course of the examination.

The following answers were rated as correct:

- **post-operative infection**
- **wound infection**
- **pain after extraction**
- **dolor post extractionem**
- **alveolar osteitis**
- **dry socket**
- **maxillary sinusitis**
- **acute maxillary sinusitis**
- **oro-antral communication**
- **OAC**
- **oro-maxillary sinus perforation**

Question 2:

What is your next step?

Correct answers:	clinical examination, extra-oral examination, extra-oral assessment, extra-oral finding, intra-oral examination, intra-oral assessment, intra-oral finding, inspection and palpation, probing of the alveole with a myrtle leaf probe, nose blowing test
Distractors:	X-ray, X-ray image, radiography, radiographic diagnostics, dental film, dental film image, OPG, orthopantomogram, CBCT, cone beam computed tomography, CT, computed tomography, MRI, magnetic resonance imaging, imaging, antibiotic therapy, prescribing antibiotics without examination, extraction, additional extractions

Feedback:

In the next step, you carry out a clinical examination including extra-oral and intra-oral assessments in order to confirm your suspected diagnosis.

Detailed feedback on question 2

Your diagnostic procedure should begin with a clinical examination of the patient, including both extra-oral and intra-oral assessments. The extra-oral assessment should comprise a visual inspection with particular attention to swellings in the right maxillary region as well as checking sensitive facial innervation and physiological mouth opening. Afterwards, the intra-oral assessment should include the examination of the extraction wound (paying attention to necrotic tissue areas and signs of inflammation) as well as excluding damage to neighboring teeth or tissues. In addition, probing of the alveole with a myrtle leaf probe and a nose blowing test are indicated in order to identify a potential oro-antral communication. If purulent secretion is leaking from the extraction alveole, this may be an indication of maxillary sinusitis.

Following the clinical examination, the justifying indication for further diagnostic measures can be made.

<u>The following answers were rated as correct:</u>

- **clinical examination**
- **extra-oral examination**
- **extra-oral assessment**
- **extra-oral finding,**
- **intra-oral examination**
- **intra-oral assessment**
- **intra-oral finding**
- **inspection and palpation**
- **probing of the alveole with a myrtle leaf probe**
- **nose blowing test**

Question 3:

During your clinical examination, you notice slight redness and palpation sensitivity in the right upper cheek region (canine fossa). The right infraorbital nerve is tender on pressure but sensitivity is not affected. The extraction socket of tooth 16 is also reddened and shows insufficient wound healing. The applied nose blowing test is positive (i.e. air bubbles appear in the area of the socket) and purulent secretion is leaking from the extraction alveole.

Which further diagnostic step is indicated now?

Correct answers:	Radiological examination, X-ray, radiography, radiographic diagnostics, radiographic overview image, radiographic assessment of the maxillary sinuses and surrounding structures, X-ray of the paranasal sinuses, paranasal sinus image, PNS image, orthopantomogram, orthopantomograph, orthopantomography, OPG, OPT
Distractors:	dental film, dental film image, CBCT, cone beam computed tomography, CT, computed tomography, MRI, magnetic resonance imaging, right-side bitewing image, curettage of the extraction socket

Feedback:

Your next diagnostic step should be a radiological examination by means of an orthopantomogram.

Detailed feedback on question 3

In the present case, the clinical examination should be followed by a radiological examination of the surrounding structures of the extraction region. In particular, the maxillary sinuses should be examined for shadows, as maxillary sinusitis is suspected. In order to search for the focus of infection, using an orthopantomogram (OPG) is indicated. This radiographic image provides a good assessment of the neighboring structures with a comparatively low radiation exposure.
A CBCT or CT scan is not initially indicated in this case, as the OPG generally provides a sufficient assessment of the maxillary sinuses. However, for further questions (that cannot be adequately answered using an OPG) a CBCT scan may be indicated.

The following answers were rated as correct:
- **radiological examination**
- **X-ray**
- **radiography**
- **radiographic diagnostics**
- **radiographic overview image**
- **radiographic assessment of the maxillary sinuses and surrounding structures**
- **X-ray of the paranasal sinuses**
- **paranasal sinus image**
- **PNS image**
- **orthopantomogram**
- **orthopantomograph**
- **orthopantomography**
- **OPG**
- **OPT**

Question 4:

The OPG shows a complete shadowing of the maxillary sinus on the side of the previously executed molar extraction.
What is your final diagnosis based on the available findings?

Correct answers:	maxillary sinusitis, right-sided maxillary sinusitis, ipsilateral maxillary sinusitis, oro-antral communication, OAC, oro-maxillary sinus perforation
Distractors:	abscess, periapical abscess, residual cyst, osteomyelitis, apical periodontitis, fracture

Feedback:

Consequently, your final diagnosis is maxillary sinusitis due to a persisting oro-antral communication.

Detailed feedback on question 4

In summary, the clinical symptoms described by the patient as well as the positive nose blowing test as well as the probing of the extraction alveole with a myrtle leaf probe, the diagnosis of an oro-antral communication (OAC) can be confirmed.
As the OAC has persisted for more than 24 hours, it can be assumed that the maxillary sinus is bacterially infected. This is supported by the leakage of purulent secretion during the nose blowing test. Finally, the OPG shows a shadow in the maxillary sinus on the extraction side and thus confirms the diagnosis of right-sided maxillary sinusitis resulting from the connection between the sinus and the bacterial flora of the oral cavity.

The following answers were rated as correct:

- **maxillary sinusitis**
- **right-sided maxillary sinusitis**
- **ipsilateral maxillary sinusitis**
- **oro-antral communication**
- **OAC**
- **oro-maxillary sinus perforation**

Attachment 1 to Becker T, Ackermann MA, Sennhenn-Kirchner S. *Formative key feature examinations as innovative teaching approach in dental education: A project report.* GMS J Med Educ. 2024;41(4):Doc39.
DOI: 10.3205/zma001694

Question 5:

Which initial therapeutic approach is indicated in the current situation?

Correct answers:	conservative treatment of maxillary sinusitis, conservative treatment of acute inflammation, conservative therapy, regular irrigation of the maxillary sinus, regular irrigation of the maxillary sinus every two days, antibiotic therapy, antibiotic treatment, decongestant nasal spray
Distractors:	plastic closure of the oro-antral communication, Rehrmann technique (plastic closure of oro-antral communication), curettage of the extraction socket

Feedback:

First, you should initiate conservative treatment of the acute inflammation. This includes regular irrigations of the maxillary sinus as well as an antibiotic therapy.

Detailed feedback on question 5

Due to the clear signs of inflammation, the first therapeutic step is to treat the acute infection of the maxillary sinus. Regular irrigations (every two days) of the affected maxillary sinus through the extraction socket are indicated for this purpose. In addition, antibiotic therapy should be initiated in combination with a decongestant nasal spray. With this pre-treatment, a sufficient drainage of the maxillary sinus via the natural ostium can be achieved. When the acute inflammation has subsided (i.e. when only clear secretion leaks out during irrigation), further treatment of the intra-oral situation can be initiated.

The following answers were rated as correct:

- **conservative treatment of maxillary sinusitis**
- **conservative treatment of acute inflammation**
- **conservative therapy**
- **regular irrigation of the maxillary sinus**
- **regular irrigation of the maxillary sinus every two days**
- **antibiotic therapy**
- **antibiotic treatment**
- **decongestant nasal spray**

Attachment 1 to Becker T, Ackermann MA, Sennhenn-Kirchner S. *Formative key feature examinations as innovative teaching approach in dental education: A project report.* GMS J Med Educ. 2024;41(4):Doc39.
DOI: 10.3205/zma001694

Question 6:

After 10 days, the patient consults you again. She reports taking the antibiotic and using nasal spray regularly as discussed. She also regularly rinsed the affected maxillary sinus and, finally, only clear fluid came out of her nose. Pain symptoms and signs of inflammation have subsided.

What is your next therapeutic step?

Correct answers:	surgical closure of oro-antral communication, plastic closure of oro-antral communication, closure of oro-maxillary sinus perforation, Rehrmann flap technique, rotational flap technique, placement of a PTFE membrane, PTFE membrane
Distractors:	wait and see, no further treatment after the inflammation subsides, curettage of the extraction socket, removal of bone tissue in the alveolar region, extraction

Feedback:

Following the treatment of the acute inflammation, the OAC has to be surgically closed. This can be done, for example, by using a Rehrmann flap technique or a rotational flap technique or by inserting a PTFE membrane.

Detailed feedback on question 6

After the inflammation of the maxillary sinus has subsided due to conservative pre-treatment, the next therapeutic step is the surgical treatment of the oro-antral communication. Its surgical closure can be carried out by using a so-called Rehrmann flap technique or a rotational flap technique or by inserting a PTFE membrane.

The following answers were rated as correct:

- **surgical closure of oro-antral communication**
- **plastic closure of oro-antral communication**
- **closure of oro-maxillary sinus perforation**
- **Rehrmann flap technique**
- **rotational flap technique**
- **placement of a PTFE membrane**
- **insertion of a PTFE membrane**
- **PTFE membrane**
